# Alterations in vaginal microbiota in uterine fibroids patients with ultrasound-guided high-intensity focused ultrasound ablation

**DOI:** 10.3389/fmicb.2023.1138962

**Published:** 2023-04-17

**Authors:** Ping-Ping Zhang, Xue-Ping He, Wen Tang, Han-Wei Chen, Yuan-Yuan Han

**Affiliations:** ^1^Department of Radiology, Panyu Central Hospital, Guangzhou, China; ^2^Department of Radiology, Guangdong Women and Children Hospital, Guangzhou, China; ^3^Department of Radiology, Panyu Health Management Center (Panyu Rehabilitation Hospital), Guangzhou, China; ^4^School of Life Sciences, South China Normal University, Guangzhou, China; ^5^Medical Imaging Institute of Panyu, Guangzhou, China

**Keywords:** uterine fibroids, high-intensity focused ultrasound, vaginal microbiota, 16SrRNA sequencing, diversity

## Abstract

**Introduction:**

Vaginal microbiota dysbiosis is closely related to diseases of the vagina and uterus. Uterine fibroids (UF) are the most common benign neoplasms of the uterus, and increased diversity in vaginal microbial of UF patients. High-intensity focused ultrasound (HIFU) is effective invasive treatment for fibroids in women who are not good surgical candidates. Whether HIFU of uterine fibroids will cause the change in vaginal microbiota has not been reported. We aimed to investigate the vaginal microbiota of UF patients with/without HIFU treatment using 16S rRNA gene sequencing.

**Methods:**

Vaginal secretions were collected from 77 UF patients (pre-operative and post-operative) and were used for comparative composition, diversity, and richness analyses of microbial communities.

**Results:**

The microbial α-diversity was significantly lower in the vaginal of UF patients with HIFU treatment. The relative abundance of some pathogenic bacteria of UF patients with HIFU treatment were significantly decreased in the bacterial phylum and genus level. *Proteobacteria* were found to be significantly upregulated as a biomarker in the HIFU treatment group in our study.

**Conclusion:**

These findings might confirm the effectiveness of HIFU treatment from the point of view of microbiota.

## Introduction

Uterine fibroids are the most common neoplasms of the uterus, affecting more than 70% of women by the onset of menopause in the world (Baird et al., 2003). Symptoms associated with uterine fibroids include abnormal menstrual bleeding, pelvic pressure or pain, urinary incontinence or retention, and constipation (Khan and Partin, 2004; Zimmermann et al., 2012), depending on the size and location of the myoma. Due to the abnormal shape of the uterine cavity and pressure of the fallopian tube, uterine fibroids are often related to impaired fertility, pregnancy complications or loss, and poor obstetrical prognosis (Cook et al., 2010; Ciavattini et al., 2015), which bring a heavy burden to women of childbearing age. Uterine fibroids thus have a profound impact on health care costs worldwide.

Vagina is an important and complex ecosystem, mainly dominated by lactic acid bacteria, but also contains a small number of fungi and parasites (Gupta et al., 2019). In adult women, the vaginal microbiota accounts for 9% of the total bacterial load, characterized by high stability and low richness and diversity indexes (Sirota et al., 2014). *Lactobacillus* are the dominant bacteria in the vagina of healthy women of childbearing age (Ravel et al., 2011). The lactic acid they produce maintains the low pH of the vaginal environment, inhibits the growth of harmful bacteria, and keeps the microecology in a relatively balanced state (Gajer et al., 2012). The balance of vaginal microbiota exerts a beneficial influence on fertility, conception, and healthy pregnancy (Younes et al., 2018). When uterine fibroids occur, the relative abundance of vaginal lactic acid bacteria increases (Moore et al., 2021). So far, there are few studies on the relationship between vaginal microbiota and uterine fibroids.

Currently, available non-medical treatments for UF include interventional radiology and surgical procedures. Surgical management options include myomectomies and hysterectomies (Giuliani et al., 2020). The re-intervention rate of myomectomies is very high—50% of women at 5 years (Fedele et al., 1995). Hysterectomy is a commonly recommended option for women with symptomatic UF, but it is unsuitable for patients who retain the uterus. Uterine artery embolization (UAE) and high-intensity focused ultrasound (HIFU) are effective invasive treatments for fibroids in women who are not good surgical candidates. UAE is limited because of its severe side effects (Braude et al., 2000). HIFU uses high-intensity transabdominal ultrasound waves to induce fibroid coagulative necrosis and regression (Lee and Yu, 2016). Whether HIFU of uterine fibroids will cause the change in vaginal microbiota has not been reported. Therefore, the purpose of this study was to investigate the vaginal microbial composition of uterine fibroids by 16SrRNA sequencing and to evaluate the effect of high-intensity focused ultrasound on vaginal microorganisms in patients with uterine fibroids.

## Materials and methods

### Criteria for recruitment of the research subjects and ethical approval

Patients with uterine fibroids who underwent HIFU in Guangzhou Panyu Central Hospital from July 2021 to January 2022 were selected and divided into two groups: the pre-operative group and the post-operative group. All patients were less than 45 years of age. The inclusion criteria for HIFU operation were patients who (1) needed operative symptomatic uterine fibroids; (2) could lie prone for more than 1 h and communicate with nurses or doctors during the operation; and (3) had uterine fibroids with a maximum diameter smaller than 15 cm. Those who are allergic to MRI contrast medium and those who are claustrophobic were excluded. All patients were confirmed that the uterine cavity was normal by hysteroscopy and the vaginal secretions were normal under the microscope. Patients with allergy-related symptoms, bacterial vaginitis, vulvovaginal candidiasis, trichomonal vaginitis, Chlamydia trachomatis, Ureaplasma urealyticum, Neisseria gonorrhoeae infection, or other subjective vaginal symptoms such as vaginal pruritus and abnormal secretions were excluded. Patients who received any antibiotics (oral or topical) or vaginal lavage or had sex within 3 days before sample collection were also excluded. At last, 32 samples from the pre-operative group and 45 samples from the post-operative group were collected.

The study was approved by the Ethics Committee of Guangzhou Panyu Central Hospital. This study was conducted by Guangzhou Panyu Central Hospital as a single center. All participants provided written consent and permitted access to medical records to obtain their related clinical information and vaginal specimens.

### Sample processing

Vaginal secretions were collected on the day of HIFU before the operation. The samples were fully sampled with aseptic cotton swabs and placed in 2 ml cryopreservation tubes, then transferred to a –80°C refrigerator within 20 min for follow-up 16SrRNA gene sequencing (Guangdong Magigene Biotechnology Co., Ltd., Guangzhou, China).

### Total DNA extraction and 16S rRNA sequencing

Deoxyribonucleic acid extraction and purification were conducted with a MagaBio Soil/Feces Genomic DNA Purification Kit (Bioer, Hangzhou, China) according to the manufacturer’s instruction. The V4 region of the 16S rRNA gene was amplified by PCR with the universal primers 515F (5-GTGCCAGCMGCCGCGGTAA-3) and 806R (5-GGACTACHVGGGTWTCTAAT-3) using a TaKaRa Premix Taq^®^ Version 2.0 (TaKaRa Biotechnology Co., Dalian, China) on a BioRad S1000PCR instrument (Bio-Rad Laboratory, Hercules, CA, USA). The PCR system was 50 μl in total, and it contained 25 μl 2 × Premix Taq, 1 μl of forward primer, 1 μl of reverse primer, 50 ng of template DNA, and double-distilled water (ddH2O) to make up the total volume. PCR was performed using the following conditions: 5 min of denaturation at 94°C, 30 cycles of denaturation at 94°C for 30 s, annealing at 52°C for 30 s, and elongation at 72°C for 30 s, and a final extension at 72°C for 10 min. The 16S rRNA gene amplicons were purified and used for sequencing library construction according to the manufacturer’s instructions for the NEBNext ^®^ Ultra™ II DNA Library Prep Kit for Illumina ^®^ (New England Biolabs, Ipswich, MA, USA). The constructed amplicon library was sequenced using the Illumina Nova 6,000 platform.

### Microbiota analysis

Fastp (an ultra-fast all-in-one FASTQ preprocessor, version 0.14.1)^1^ was used to perform sliding window quality clipping (-W 4 -M20) on paired-end raw reads data as described previously (Chen et al., 2018). According to the primer information at the beginning and end of the sequence, cutadapt soft^2^ was used to remove the primers to obtain paired-end clean reads after quality control (Martin, 2011). For paired-end clean reads, according to the overlaping relationship between PE reads, usearch-fastq_mergepairs (V10)^3^ was used to filter the unmatched Tags to obtain the raw tags (Greay et al., 2019). Then raw tags sequences were filtered to get the clean tags. UPARSE was used to classify high-quality sequences into operational taxonomy units (OTUs) at a cutoff of 97% identity, then each OTU was annotated by the SILVA ribosomal RNA gene database project (Quast et al., 2013) after merging reads *via* overlaps. Both DADA2 and Deblur in QIIME2 were used to denoise amplicon sequence (Callahan et al., 2016; Amir et al., 2017).

Bacterial richness and diversity across the samples were calculated using the index of Chao 1. The principal-component analysis (PCA) was performed by R software prcomp function. The vegan software package of R software was used to combine the bray_curtis algorithms and hclust functions for heat map cluster analysis. Linear discriminant analysis (LDA) was performed using LEfse software. LEfSe was used to identify biomarkers of microbiomes in vaginal samples of the pre-operative group and the post-operative group at multiple levels in datasets, grade biomarkers based on statistical significance, and visualize the results using taxonomic bar charts (Segata et al., 2011). The Illumina sequences reported here have been deposited in the NCBI Sequence Read Archive^4^ under accession number PRJNA926005.

## Statistical analysis

GraphPad Prism V.8.0 (San Diego, CA, USA) was used for all analyses and preparation of graphs. The results of vaginal microbiome diversity indices were expressed as the median value and analyzed using the non-parametric Wilcoxon test. The relative abundance of a microbe in a sample was calculated as the read count normalized against the total reads in that sample. The relative abundance value for each genus was depicted as mean ± SE. Statistical analyses were performed using a two-tailed non-parametric Wilcoxon test to evaluate the significance of differences in microbial taxa between groups, with FDR correction. To identify biomarkers in the vaginal microbiota associated with HIFU operation, the non-parametric Kruskal–Wallis test is used to detect species with significant abundance differences between different groups, and then the group Wilcoxon test is used to judge the difference between the two groups, and finally LDA is used to reduce the dimension and evaluate the impact size of significantly different species (LDA Score, the filter value of LDA Score to 2 by default), and the result is the biomarker in each group. Differences with a *P*-value of < 0.05 were considered significant.

## Results

### Diversity of the vaginal microbiota

**FIGURE 1:**
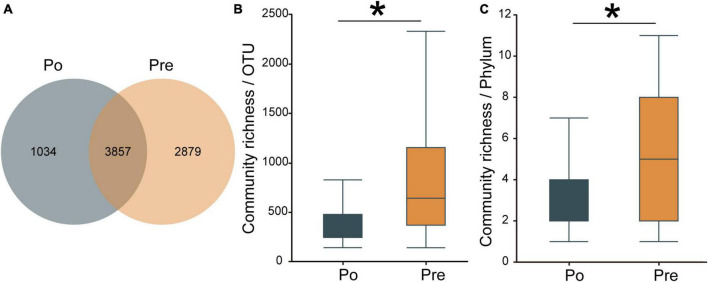
Diversity of the vaginal microbiota. Venn diagrams for overlap between the pre-operative observed OTUs versus post-operative group observed OTUs. Pre contained 6,736, and Po contained 4,891, among which 3,857 were shared between the two groups **(A)**. The α-diversity of the microbiota was calculated in OTUs level **(B)** and phylum level **(C)** by the species richness index. The pre-operative group (Pre); post-operative group (Po). *p < 0.05.

A total of 77 samples were analyzed by 16 S rRNA gene sequencing to investigate the vaginal microbiota, including 32 samples from the pre-operative group and 45 from the post-operative group. In total, 6,786,912 reads were obtained from these 77 samples, on average, 88,142 reads per sample. As the number of samples increased, the Pan/Core curve of the operational taxonomic unit (OTU) number was almost a straight horizontal line, demonstrating that the samples were sequenced with enough depth in this study (see Supplementary Figure 1).A total of 7,770 OTUs were obtained in the two groups. The pre-operative group contained 6,736, and the post-operative group contained 4,891, among which 3,857 were shared between the two groups (Figure 1A). The number of OTUs in the pre-operative group was much larger than that in the post-operative group, indicating that the microbial composition of the pre-operative group was more abundant. The α-diversity of the microbiota was calculated by the species richness index (OTU level: 893 ± 680 for the pre-operative group and 566 ± 656 for the post-operative group, *P* = 0.03999, Figure 1B; Phylum level: 5.64 ± 4.21 for the pre-operative group and 3.97 ± 3.36 for the post-operative group, *P* = 0.03822, Figure 1C). A rarefaction curve of the observed OTUs against sequences per group was plotted to determine the efficiency of the sequencing process (see Supplementary Figure 2). Based on the observation that the curve of rarefied counts should plateau if the sample is close to saturation (Rodriguez-R and Konstantinidis, 2014), the results provide a measure of the depth of samples in our study. These results demonstrated that the microbial diversity in the vaginal environment was significantly higher in the pre-operative than in the post-operative.

### Composition of the vaginal microbiota

**FIGURE 2:**
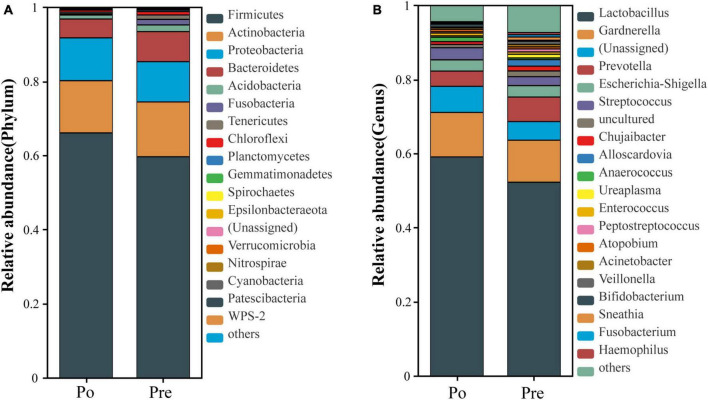
Composition of the vaginal microbiota. Comparison of the average abundance of each bacterial phylum in the pre-operative and post-operative groups **(A)**. Comparison of the average abundance of each bacterial genus in the pre-operative and post-operative groups **(B)**.

The community composition of each sample, representing the bacterial phylum and genus level ranking for major OTU, is depicted in Figure 2. The taxonomic classification at the phylum level showed similar patterns in the two groups, dominated by Firmicutes, Actinobacteria, Proteobacteria, Bacteroidetes, and *Acidobacteria*. However, the relative abundance of Proteobacteria was significantly increased in the post-operative group (*P* = 0.01753); the relative abundances of *Acidobacteria*, *Fusobacteria*, *Chloroflexi, Planctomycetes, Gemmatimonadetes, Spirochaetes, Verrucomicrobia, Nitrospirae, Cyanobacteria* were significantly decreased in the post-operative group (*P* < 0.05; Figure 2A). At the genus level, genera with the top 20 abundances are shown in Figure 2B. Among them, the abundances of *Lactobacillus*, *Gardnerella*, *Prevotella, Escherichia-Shigella*, and *Streptococcus* were higher than 1% in both the pre-operative and the post-operative groups, while those of *Chujaibacter, Alloscardovia*, and *Ureaplasma* were >1% only in the pre-operative group. There were four significantly different genera between the two groups. The relative abundances of *Chujaibacter, Acinetobacter, Fusobacteriumi*, and *Haemophilus* were decreased considerably in the post-operative group (*P* < 0.01; Figure 2B).

### Distribution of the vaginal microbiota in all the samples

**FIGURE 3:**
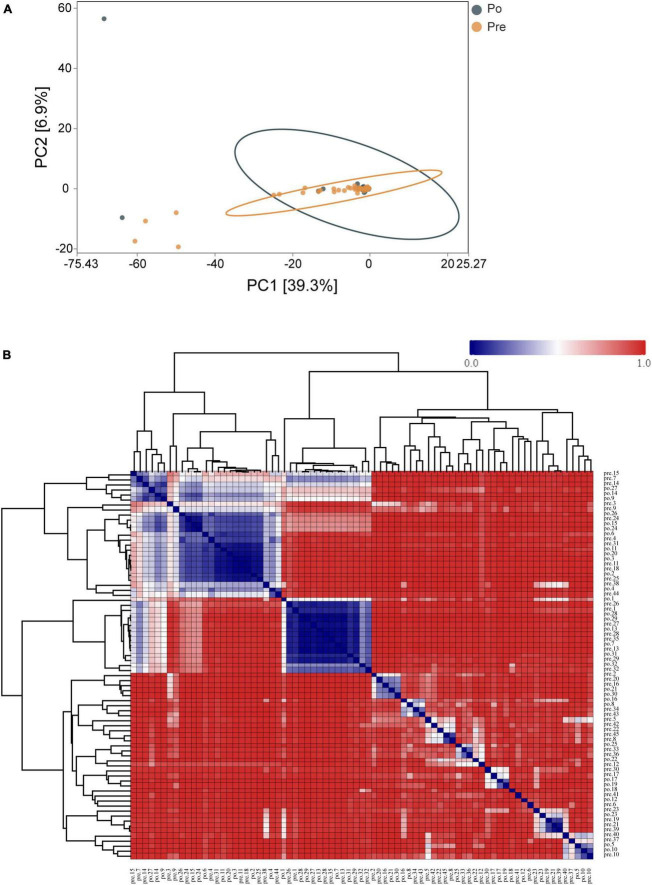
Distribution of the vaginal microbiota in all the samples. PCA was applied to demonstrate the distribution of the vaginal microbial communities in the samples **(A)**. Each symbol represents a sample (dark green, Po; brown, Pre); the variance explained by the PCs is indicated in parentheses on the axes. Heatmap based on the hierarchical clustering solution (Bray–Curtis’s distance metric of the 77samples) **(B)**. Rows and columns represent the 77 samples. The lower number represents more remarkable similarity in bacterial microbiota between samples in the heatmap. Note: the pre-operative group (Pre); post-operative group (Po).

Principal-component analysis (PCA) was applied to illustrate the distribution of the microbial community in the samples at genus level (Figure 3A). The first principal component (PC1, *x*-axis) explains 39.3% of the variation in the data, while the second principal component (PC2, y-axis) decreases total explained variation to 6.9%. This, along with the Analysis of Similarity in the composition and structure (ANOSIM, *R* = 0.00868, *P* = 0.268), indicates the two groups are similar, which presents the lack of dissimilarity in RNA expression between the pre-operative and post-operative at genus level. Moreover, a clustered heatmap of the beta-diversity index displaying bray-Curtis dissimilarity at the OTU level between individual samples was shown in Figure 3B.

### Vaginal microbiota associated with HIFU operation

**FIGURE 4:**
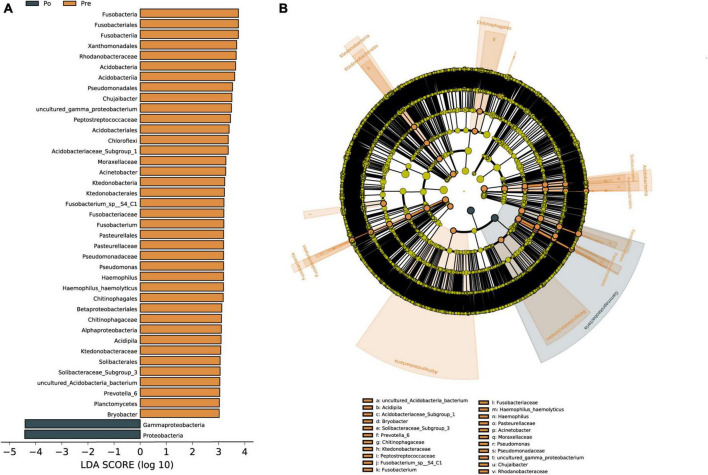
Vaginal microbiota associated with HIFU operation. Linear discriminant analysis (LDA) effect size (LEfSe) analysis of vaginal microbiota changes following HIFU treatment. Linear discriminant analysis of the differentially abundant genera, which indicated their contribution to group differentiation. Cladogram using LEfSe method indicating the phylogenetic distribution of vaginal microbiota associated with patients with pre-operative group (brown) and post-operative (dark green) **(A)**. The phylogenetic tree shows LDA scores calculated for differences in genus-level abundance between the pre-operative group and post-operative group **(B)**. The brown bars indicate that the genus was more abundant in the pre-operative group. The dark green bars indicate that those genera were more abundant in the post-operative group.

Linear discriminant analysis effect size (LEfSe) was used to identify vaginal microbiota, which differentiated vaginal microbiota in the pre-operative group from microbiota in the post-operative group (Figure 4A). The vaginal microbiomes of the post-operative group were characterized by a preponderance of *Proteobacteria* and *Gammaproteobacteria*, whereas the microbiomes were characterized by a preponderance of *Acidobacteria*, *Bacteroidia, Ktedonobacteria, Clostridia, Fusobacteria, Alphaproteobacteria, Gammaproteobacteria* and *Proteobacteria* in the pre-operative group (Figures 4A, B). *Gammaproteobacteria* is the largest and the most diverse group of Proteobacteria. The microbial biomarker (*Proteobacteria*) showed a significant increase in abundance in the post-operative group than the pre-operative group (*P* < 0.05). Overall, *Proteobacteria* were found to be significantly upregulated as biomarkers in the HIFU treatment group in our study.

## Discussion

Microbiota is symbiotic with human and affects our health, and its role has been paid more and more attention. Compared with the study of microbiota in the gastrointestinal tract, oral cavity, and skin, the understanding of microflora in the female reproductive tract is just beginning. The relationship between vaginal microbiota and infectious diseases, adverse obstetrical outcomes, and gynecological tumors was studied. The vaginal microbiota is one of the critical determinants of vaginal health, together with the endocrine system, vaginal immune microenvironment, and vaginal epithelial cells, which constitute the defense barrier of vaginal health (France et al., 2022). The abundance and diversity of bacteria were significantly higher in patients with gynecological diseases (uterine leiomyoma, adenomyosis, and endometrial polyps) than in those without (Chen et al., 2017). As observed above, an increase in vaginal microbial diversity in UF patients in the pre-operative group was observed in this study. Similarly, it was found that the intestinal flora of patients with UF before the operation was higher than that after the abdominal hysterectomy (Wang et al., 2020). The treatment of HIFU significantly decreased the abundance and diversity of the vaginal microbiome in UF patients.

From our results, the dominant bacteria in this study are the same as those in previous studies based on 16SrRNA gene sequences (Huang et al., 2019; Yao et al., 2021). *Lactobacillus* was the most abundant vaginal bacteria, representing 60∼90% of vaginal bacteria in healthy women (Kim et al., 2009; Ravel et al., 2011). However, other bacterial genera have been observed as part of the normal vaginal bacteria, including *Atopobium*, *Streptococcus*, *Anaerococcus*, *Peptoniphilus*, *Prevotella*, *Gardnerella*, *Sneathia*, *Mobiluncus*, and *Finegoldia* (Ceccarani et al., 2019; Fu et al., 2020; Yao et al., 2021). From our results, *Lactobacillus* is the predominant bacteria in the two groups, followed by *Gardnerella*, *Prevotella*, Escherichia-Shigella, and *Streptococcus*. However, only the relative abundances of *Chujaibacter*, *Acinetobacter*, *Fusobacterium*, and *Haemophilus* were significantly decreased in the post-operative group. Acinetobacter is a multidrug-resistant bacterium that is usually a commensal pathogen, but might lead to pneumonia, fever, and septicemia (Aivazova et al., 2010). Fusobacterium, a gram-negative spindle-shaped obligate anaerobe, is ubiquitous in the human mouth (Han et al., 2010), also colonized in vaginal with bacterial vaginosis, amniotic fluid infections and preterm labor (Agarwal et al., 2020). Haemophilus can cause different illnesses involving breathing, bones and joints, and the nervous system. An intramyometrial abscess caused by Haemophilus influenza has been reported (Ambler et al., 2010). The treatment of HIFU significantly decreased the above pathogen.

Principal-component analysis demonstrated similarities in the visual coordinate data, along with the Analysis of Similarity in the composition and structure (ANOSIM, *R* = 0.00868, *P* = 0.268), indicates the two groups are similar between the pre-operative group and the post-operative group. The PCoA analysis of microbiota composition indicated that there was a distinct clustering pattern between samples from UF individuals and healthy controls (Mao et al., 2022). The NMDS analysis results (see Supplementary Figure 3) were not similar to those of PCoA and the value of stress = 0.167 (<0.2). Because HIFU treatment just shrink the fibroids but didn’t eliminate them (Liu et al., 2021), the vaginal microenvironment has not changed markedly. Although they did not show a stable difference in the statistical analysis of β-diversity in genus level, there was a significant difference in the statistical analysis of α-diversity in OTUs and phylum level, so there was a difference in microbial diversity in general.

Linear discriminant analysis was conducted to evaluate the influence of biomarkers on the pre-operative group and the post-operative group based on the LDA score. In the phylum, *Gammaproteobacteria* and *Proteobacteria* were enriched in the post-operative group. *Gammaproteobacteria* is the largest and the most diverse group of Proteobacteria. The phylum *Gammaproteobacteria* and lower taxa, including the *Xanthomonadales*, *Pseudomonadales*, and *Betaproteobacteriales*, were enriched in the pre-operative group. The *Gammaproteobacteria* comprise several medically important groups of bacteria, such as the *Enterobacteriaceae*, *Vibrionaceae*, and *Pseudomonadaceae* (Lu et al., 2017). Increased prevalence of *Proteobacteria* is a potential diagnostic signature of dysbiosis and disease risk (Shin et al., 2015). The level of Proteobacteria in the gut microbiota of uterine fibroids group was significantly lower than those in the blank control group, and Proteobacteria was negatively correlated with the incidence of uterine fibroids (Zhang et al., 2019). A recent study shows that Ejiao, and turtle carapace glue, may play a certain therapeutic role in uterine fibroids by reducing the ratio of Firmicutes and Bacteroides and increasing the relative level of Proteobacteria in the gut microbiota (Qiu et al., 2022). Svensson et al. (2021) reported that the abundance of 12 bacteria in the classes *Bacilli, Bacteroidia, Clostridia, Coriobacteriia, and Gammaproteobacteria* strongly varied between stool samples of endometriosis patients and healthy individuals. So far there has been no literature reporting *Proteobacteria* as a biomarker of vaginal microbiota in patients with uterine fibroids, *Proteobacteria* were found to be significantly upregulated as a biomarker in the HIFU treatment group in our study. Although our present study focused on changes in microbial microbiota before and after HIFU treatment, a healthy group should also be considered. There are still some limitations in this study that need to be further explored. Firstly, the vaginal microbiota of healthy women will also be included in our studies, which may help us find some solid biomarkers for the vaginal microbiota of HIFU treatment. A correlation analysis of sex hormones and vaginal microbiota needs to be included. In addition, larger sample sizes will need to be expanded for more reliable studies in the future.

The present study provides the first evidence that treatment of HIFU for UF decreased microbial diversity and some pathogenic microbiota in the vaginal. *Proteobacteria* were found to be significantly upregulated as a biomarker in the HIFU treatment group in our study. These findings might confirm the effectiveness of HIFU treatment from the point of view of microbiota.

## Data availability statement

The datasets presented in this study can be found in online repositories. The names of the repository/repositories and accession number(s) can be found below: NCBI–PRJNA926005.

## Ethics statement

The studies involving human participants were reviewed and approved by the Ethics Committee of Guangzhou Panyu Central Hospital. The patients/participants provided their written informed consent to participate in this study.

## Author contributions

P-PZ designed and performed the experiments. X-PH and WT performed the experiments. Y-YH analyzed the data and wrote the manuscript. H-WC provided with suggestions on experimental design. All authors contributed to the article and approved the submitted version.

## Footnotes

1
https://github.com/OpenGene/fastp/

2
https://github.com/marcelm/cutadapt/

3
http://www.drive5.com/usearch

4
https://submit.ncbi.nlm.nih.gov/subs/bioproject/
