# Supplementary figures and images for: Alterations in vaginal microbiota in uterine fibroids patients with ultrasound-guided high-intensity focused ultrasound ablation
Source: Front Microbiol. 2023 Apr 17;14:1138962. doi: 10.3389/fmicb.2023.1138962 (PMC10150040; doi:10.3389/fmicb.2023.1138962)

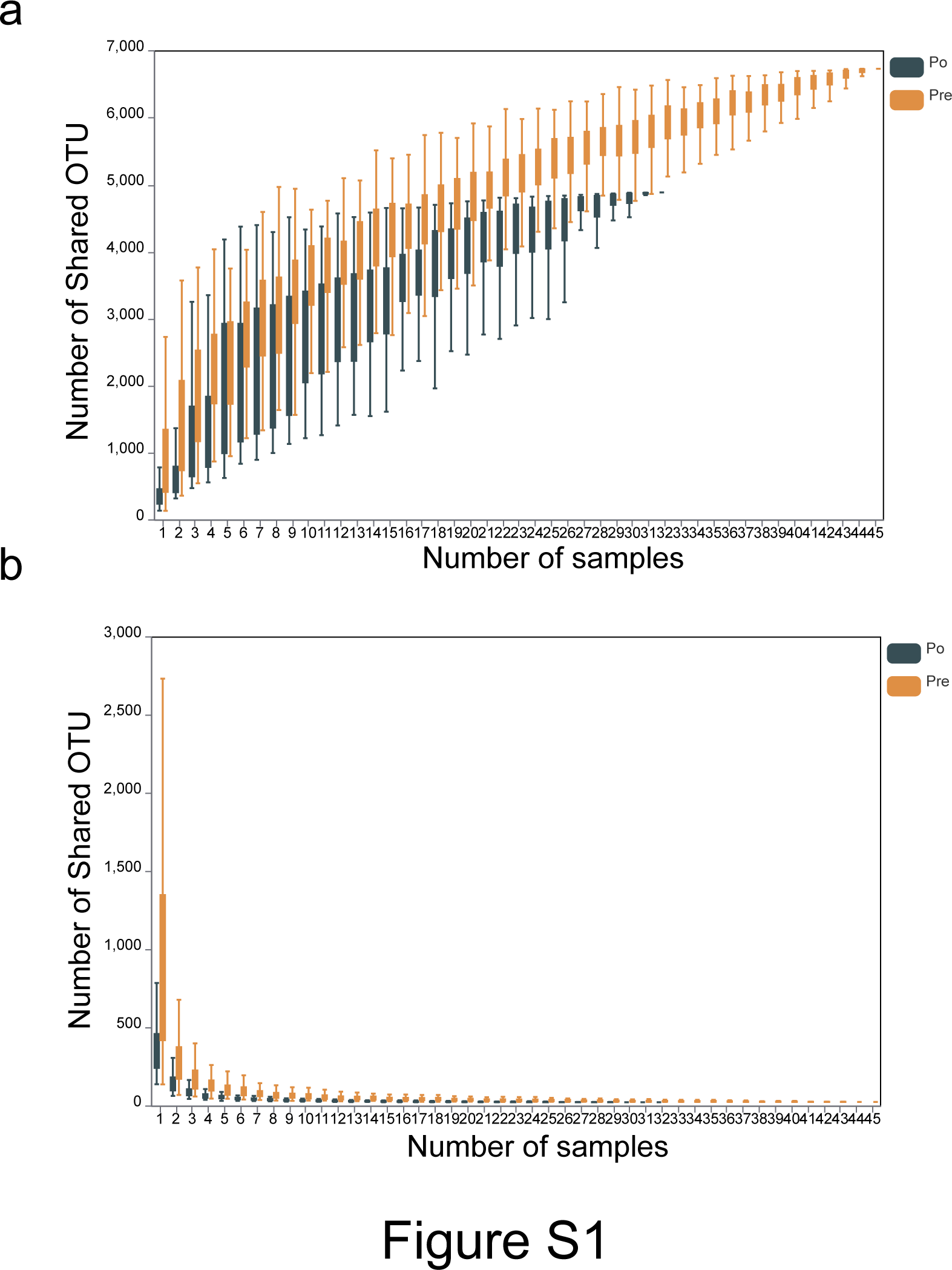

Supplement: Supplementary file 1 [file Image_1.TIF]

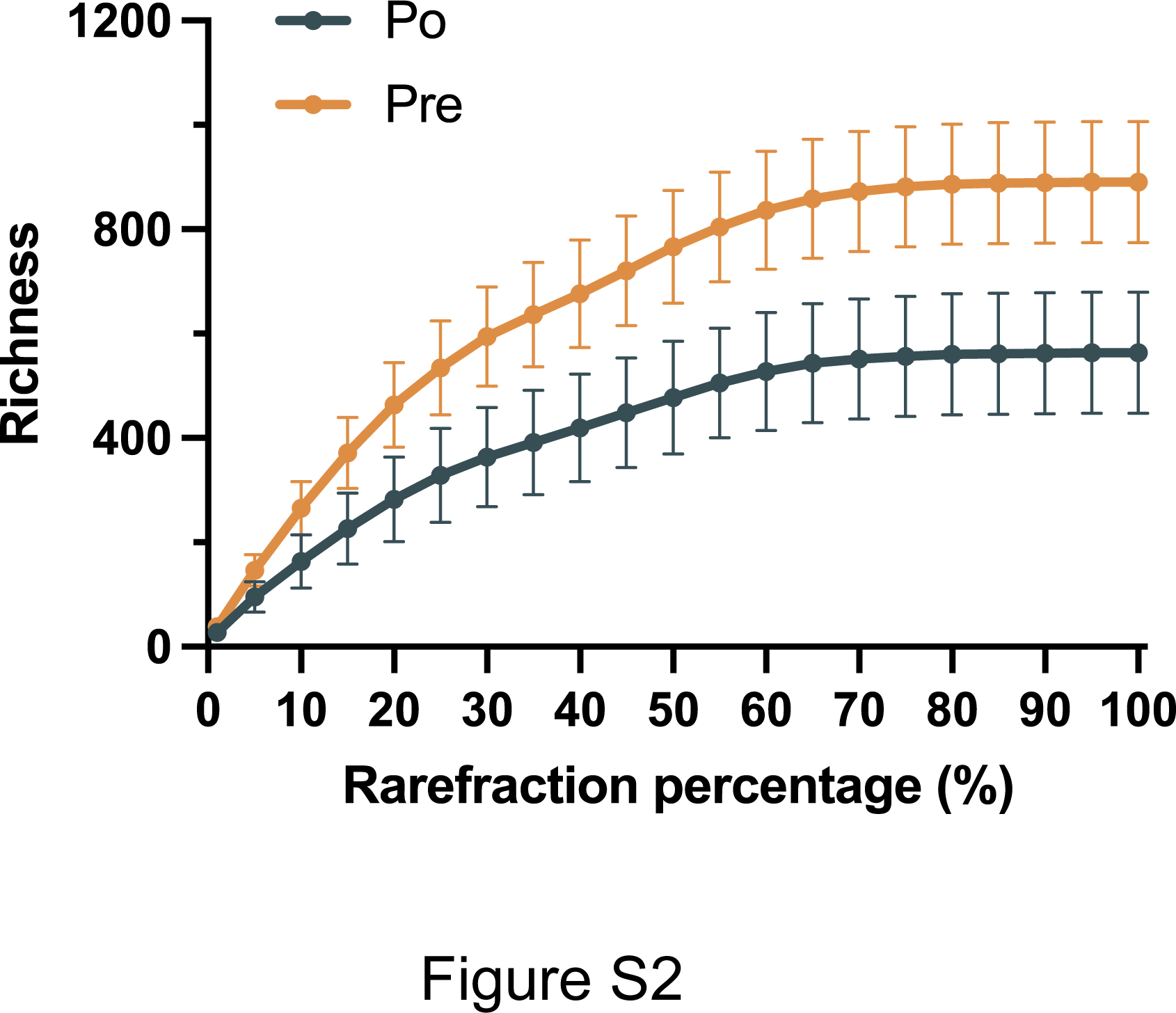

Supplement: Supplementary file 2 [file Image_2.TIF]

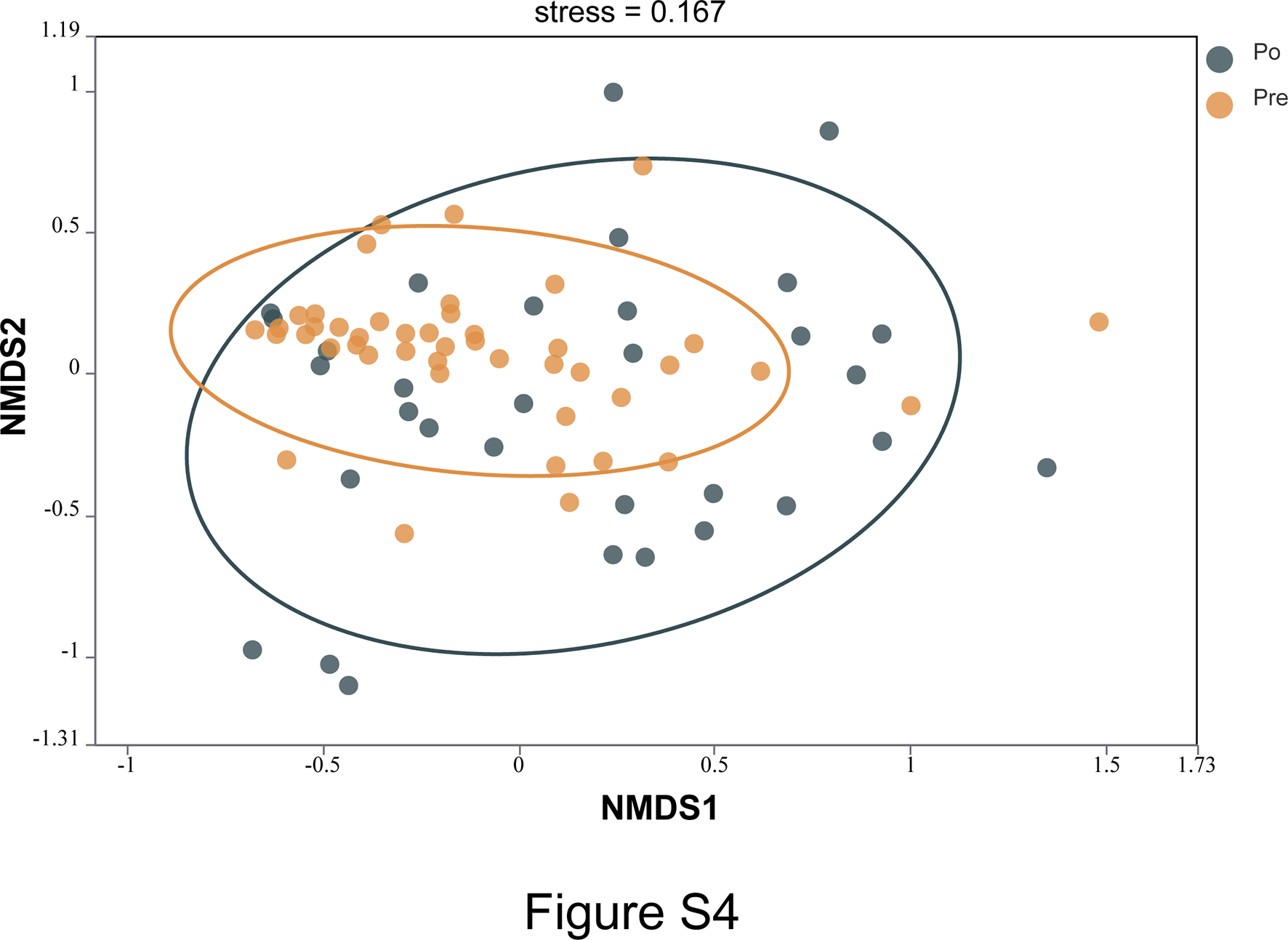

Supplement: Supplementary file 3 [file Image_3.TIF]
